# Ectomycorrhizal fungi: Potential guardians of terrestrial ecosystems

**DOI:** 10.1002/mlf2.12127

**Published:** 2024-07-31

**Authors:** Wenchen Song

**Affiliations:** ^1^ College of Life and Environmental Sciences Minzu University of China Beijing China

The tolerance of terrestrial ecosystems to anthropogenic stress and climate change has received increasing attention considering the intensification of global changes caused by human activities^1^. Improvement of the carbon sequestration capacity and ability to mitigate global changes in ecosystems, especially forest ecosystems, are simultaneously receiving increasing attention owing to the lack of sufficient effective negative emission technologies^1^, ^2^. However, considering the upper limit of the ecosystem service function of terrestrial plants, methods to further enhance the ability of terrestrial ecosystems to cope with global changes have become a topic of concern for researchers^3^, ^4^. Mycorrhizal fungi can form stable symbiotic relationships with plants and promote plant stress resistance and nutrient element utilization efficiency, thereby enhancing soil carbon sequestration^5^, ^6^, ^7^. Consequently, mycorrhizal fungi are considered crucial in enhancing the resilience of terrestrial ecosystems against global environmental change. Studying and utilizing the ecosystem service functions of mycorrhizal fungi have, therefore, become vital to cope with global changes.

Mycorrhizal fungi are commonly divided into four types (arbuscular mycorrhizal, orchid mycorrhizal, ericoid mycorrhizal, and ectomycorrhizal fungi), of which the most common and important are arbuscular mycorrhizal fungi and ectomycorrhizal fungi^8^, ^9^. Arbuscular mycorrhizal fungi have a higher diversity (approximately 72% of all mycorrhizal fungal species) and larger distribution range (approximately 70% of global terrestrial ecosystems) than ectomycorrhizal fungi, thus receiving more attention from researchers^8^, ^10^, ^11^. Over the past 24 years, according to the Web of Science, although the literature on arbuscular mycorrhizal fungi and ectomycorrhizal fungi has demonstrated an increasing trend, the literature on arbuscular mycorrhizal fungi has been far greater than that on ectomycorrhizal fungi (Figure 1A). In 2023, the literature on arbuscular mycorrhizal fungi was 5.7 times that in 2000. In comparison, the literature on ectomycorrhizal fungi increased only 2.5 times over the same period (Figure 1A). The literature on arbuscular mycorrhizal fungi was approximately 4 times that of ectomycorrhizal fungi in 2023 (Figure 1A). This seems to indicate that researchers generally believe that the ecological role of arbuscular mycorrhizal fungi is higher than that of ectomycorrhizal fungi. However, is this the case?

**Figure 1:**
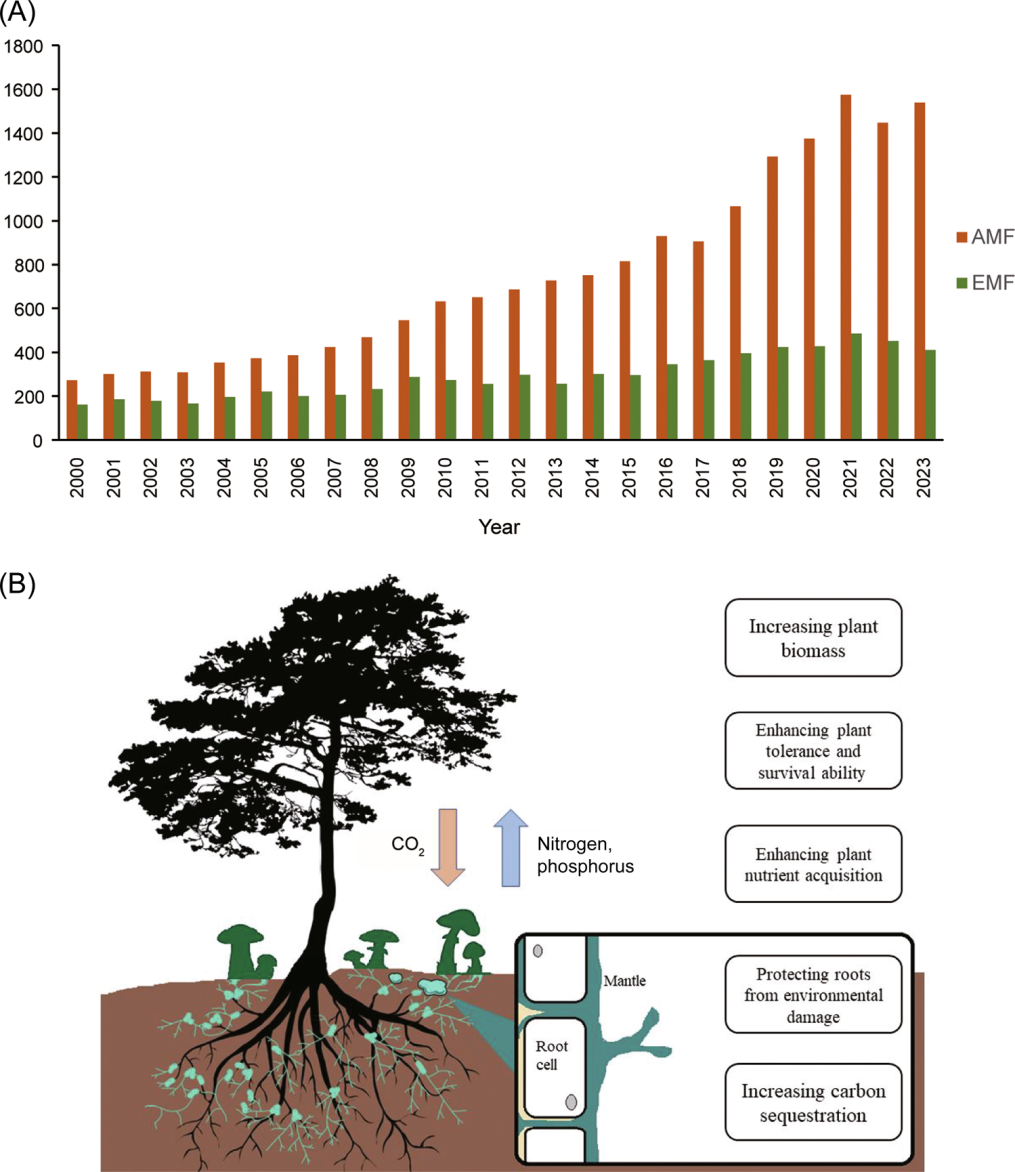
Role of ectomycorrhizal fungi. (A) Number of publications on arbuscular mycorrhizal fungi (AMF) and ectomycorrhizal fungi (EMF). Data are from the Web of Science (keywords: arbuscular mycorrhizal fungi and ectomycorrhizal fungi). (B) Role of ectomycorrhizal fungi in protecting terrestrial ecosystems and responding to global changes.

## COMPARISON OF PLANT RESISTANCE ENHANCEMENT

The biggest difference between arbuscular mycorrhizal fungi and ectomycorrhizal fungi is that the hyphae of arbuscular mycorrhizal fungi extend into the cells of the root system, while the hyphae of ectomycorrhizal fungi are encapsulated outside the cells, eventually forming a biofilm on the surface of the root system. Ectomycorrhizal fungi act as a protective armor for plant roots against pathogenic microorganisms^12^, ^13^, soil pollutants^14^, ^15^, and even nuclear radiation^16^, ^17^; this biofilm enhances the environmental resistance of plants. Moreover, such fungi facilitate the direct uptake of nitrogen and phosphorus from soil organic matter, which not only boosts the survival ability of plants under increased nutrient acquisition efficiency but also makes plants more adaptable to harsh environments, such as drought, low nutrient availability, and cold conditions^18^, ^19^, ^20^, ^21^. Ectomycorrhizal fungi also protect biodiversity in high‐latitude temperate regions through their interactions with plants^13^, ^19^, ^22^. Although arbuscular mycorrhizal fungi can improve plant nutrient acquisition efficiency, no advantage over ectomycorrhizal fungi in other aspects has been found^23^, ^24^, making them unsuitable for coping with harsh environments caused by global changes.

## COMPARISON OF CARBON SEQUESTRATION IMPROVEMENT

Fungal mycelia, especially the biofilm and fruiting body of ectomycorrhizal fungi, are primarily composed of compounds that are difficult to decompose. These microbial residues are constantly transported into the soil carbon pool, promoting underground carbon sequestration (known as the “entombing effect”)^25^. This effect of arbuscular mycorrhizal fungi, however, is not as strong as that of ectomycorrhizal fungi^24^. Ectomycorrhizal fungi are believed to directly “mine” nitrogen from organic substrates through the enzymes, which can reduce their ability to decompose litter and soil organic matter, thereby slowing down organic matter decomposition and reducing the loss of soil carbon^23^, ^24^. By contrast, arbuscular mycorrhizal fungi require the decomposition of saprophytes into inorganic matter before they can utilize soil nutrients, also resulting in some carbon loss^20^. Therefore, plants that have symbiotic relationships with ectomycorrhizal fungi can overcome nutrient limitations, enhance their tolerance to climate, and increase their biomass, thus making the ecosystem dominated by ectomycorrhizal fungi have higher biomass than that dominated by arbuscular mycorrhizal fungi^6^, ^26^, ^27^, ^28^. Plant species associated with ectomycorrhizal fungi show a biomass increase of approximately 30% for elevated CO_2_, while plant species associated with arbuscular mycorrhizal fungi show almost zero change in biomass^29^.

## SUGGESTIONS FOR THE FUTURE

In summary, compared to arbuscular mycorrhizal fungi, ectomycorrhizal fungi can effectively increase soil and biomass carbon pools, reduce soil carbon loss, and have greater climate change mitigation potential (Figure 1B). Considering the several advantages of ectomycorrhizal fungi in enhancing the capacity of terrestrial ecosystems to cope with global environmental change, the following recommendations are made:

1. More attention should be paid to the study of ectomycorrhizal fungi and current research is insufficient. Extreme climate change, for example, increasing temperatures and rainfall, impairs the growth and development of ectomycorrhizal fungi^30^, ^31^, ^32^. Therefore, future research should explore mechanisms for maintaining the competitiveness of ectomycorrhizal fungi under high temperatures and heavy rainfall.
2. Considering that ectomycorrhizal fungi are more adapted to harsh environments, such as dry, cold, and barren environments^6^, ^19^, ^21^ and because ectomycorrhizal fungi are often dominant species in the topsoil community^8^, ^33^, ^34^, the intentional introduction of ectomycorrhizal fungi during ecosystem restoration activities, such as afforestation and grass planting, could accelerate the community succession processes and enable ectomycorrhizal fungi to provide their ecosystem services and functions.
3. Globally, ecosystems dominated by ectomycorrhizal fungi are not widespread, and anthropogenic transformation of ecosystems has reduced the presence of ectomycorrhizal vegetation, with potential implications for terrestrial carbon storage^10^. Therefore, the distribution range and dominance of ectomycorrhizal fungi could be expanded to enhance their role in terrestrial ecosystems. For example, artificial inoculation or sowing could be used to establish ecosystems dominated by ectomycorrhizal fungi, which would enhance the capacity of terrestrial ecosystems to cope with global environmental change^35^.
